# A catechol-modified quaternized chitosan/PEG hydrogel for diabetic wound healing: synergistic effects of TGF-β3 delivery, angiogenesis, and antibacterial activity

**DOI:** 10.3389/fcimb.2025.1717082

**Published:** 2025-12-05

**Authors:** Xiu Yang, Zheng-Chao Zhang, Bo Liu, Yu-Nan Lu, Xue-Yi He, Hui-Dong Chen, Jia-Xun Yang, Jia-Yu He, Yi-Ren Zhu, Chang-Li Huang, Wu-Bing He

**Affiliations:** 1 Shengli Clinical Medical College of Fujian Medical University, Fuzhou, China; 2 Fuzong Clinical Medical College of Fujian Medical University, Fuzhou, China; 3 Fuzhou University Affiliated Provincial Hospital, Fuzhou, Fujian, China; 4 Department of Emergency Trauma Surgery, Fujian Trauma Medicine Center, Fujian Key Laboratory of Emergency Medicine, Fujian Provincial Hospital, Fuzhou, China

**Keywords:** diabetic wound healing, hydrogel, TGF-β3, quaternized chitosan, angiogenesis, antibacterial effect, sustained release

## Abstract

**Background:**

Diabetic foot ulcers (DFUs) represent a challenging chronic wound model, often plagued by biofilm formation that sustains inflammation and impedes healing. Transforming growth factor-beta 3 (TGF-β3) is a promising cytokine for tissue regeneration, yet its delivery within the hostile, infected wound milieu remains problematic. This study addresses the intertwined challenges of microbial resistance and healing impairment by developing a multifunctional injectable hydrogel that couples inherent antibacterial activity with sustained TGF-β3 release.

**Methods:**

A catechol-modified quaternized chitosan (QCS-Catechol) was synthesized and crosslinked with benzaldehyde-terminated 4-arm polyethylene glycol (4-arm PEG-CHO) to form the BP-QS/TGF-β3 hydrogel. Its physicochemical properties, injectability, adhesion, and mechanical strength were characterized. Antibacterial efficacy was evaluated against *Staphylococcus aureus* and *Escherichia coli*. The biocompatibility and therapeutic potential of the BP-QS/TGF-β3 hydrogel were evaluated through *in vitro* assays (CCK-8, apoptosis, hemolysis) and in a streptozotocin-induced diabetic mouse model, respectively, against controls including PBS, BP-QS (blank hydrogel), and free TGF-β3 solution. Wound closure kinetics, histology (H&E, Masson’s trichrome), and immunohistochemistry (CD31, Ki-67) were analyzed.

**Results:**

The BP-QS/TGF-β3 hydrogel demonstrated rapid gelation (~3 min), excellent injectability, robust tissue adhesion (28.5 ± 2.1 J/m²), and suitable mechanical properties. It exhibited outstanding biocompatibility and potent, broad-spectrum antibacterial efficiency (>94%). The sustained release of TGF-β3 significantly enhanced fibroblast migration and proliferation *in vitro*. In diabetic mice, the BP-QS/TGF-β3 treatment achieved the most rapid wound closure, with the lowest relative wound deficit (7.30% ± 2.76% on day 12), significantly outperforming the PBS control, BP-QS hydrogel, and free TGF-β3 solution groups (p < 0.01). Histological analyses revealed enhanced granulation tissue formation, collagen deposition (724.61 ± 60.12 μm), angiogenesis, and cell proliferation. No systemic toxicity was observed.

**Conclusions:**

The BP-QS/TGF-β3 hydrogel synergizes potent antibacterial action with sustained TGF-β3 delivery to disrupt the vicious cycle of biofilm infection and healing failure. This integrated, multidisciplinary strategy effectively targets the core pathology of diabetic wounds, offering a promising therapeutic platform for managing biofilm-infected chronic wounds.

## 1 Introduction

A common and serious complication in diabetes patients is diabetic foot ulcers (DFUs), which have a global prevalence rate of 6.3% (Zhang et al., 2017). The severity of DFUs is reflected not only in their high incidence and recurrence rates, but also in the elevated risks of amputation and mortality associated with the condition (Swaminathan et al., 2024). It is well known that diabetic wounds often remain trapped in a chronic inflammatory phase and fail to progress into the proliferation stage. Specifically, the hyperglycemic environment promotes a pro-inflammatory phenotype in macrophages, while also impairing the bactericidal and phagocytic functions of immune cells, collectively resulting in the cessation of the inflammatory period (Villa et al., 2024). Hyperglycemia and oxidative stress downregulate HIF-1/VEGF expression, impairing angiogenesis and causing localized ischemia. Additionally, although oxygen is essential for collagen production, the partial pressure of oxygen in diabetic wounds is typically lower than normal levels, thereby suppressing angiogenesis, epithelial renewal, and extracellular matrix (ECM) synthesis, all these compromise the procedure of wound recovery (Voza et al., 2024). Furthermore, cellular dysfunction in diabetic wounds encompasses a range of cell types like neutrophils, macrophages, stem cells, fibroblasts, keratinocytes, and endothelial cells. Under diabetic conditions, these cells exhibit aberrant biological behaviors, such as inhibited proliferation and differentiation, reduced migratory capacities, and compromised vascularization (McDermott et al., 2023). A high-glucose microenvironment accelerates the accumulation of advanced glycation end products, increasing vulnerability to bacterial infections, impeding the formation of blood vessels and slowing down the healing process (Yang et al., 2022a).

Transforming growth factor-beta 3 (TGF-β3), a member of the TGF-β family, is essential in the wound-healing process. Specifically, TGF-β3 regulates cell proliferation, growth, differentiation, and migration, while also stimulating fibroblasts and vascular endothelial cells to promote ECM production and accelerate vascularization (Li et al., 2023b). Additionally, TGF-β3 contributes to tissue repair by facilitating wound healing and reducing scar formation. In this context, TGF-β3 promotes the migration of fibroblasts and encourages their organized arrangement into a reticular structure, thereby supporting ECM deposition and the reconstruction of a normal dermal architecture to promote scarless healing (Wang et al., 2024). Additionally, TGF-β3 increases procollagen production in fibroblasts and significantly influences collagen synthesis and degradation. The activation of relevant signaling pathways mediates these effects, leading to an accelerated healing process for wounds (Shi et al., 2021; Zou et al., 2025).

Hydrogels, an innovative type of biomaterial, have shown great promise in healing diabetic wounds because of their outstanding biocompatibility, ability to retain moisture, and adjustable release characteristics (Zhang et al., 2024). Injectable hydrogels foster a moist environment that promotes healing by facilitating cell movement and growth, while also reducing scar formation. As wound dressings, hydrogels can soak up wound exudate, keep a moist microenvironment, and create a supportive environment for cellular functions, which boosts cell growth and movement, speeding up the healing process (Huang et al., 2022). Although significant research attention has focused on the development of novel hydrogels and their implementation in persistent diabetic ulcers, existing hydrogel systems still fall short of meeting clinical demands. For example, hydrogel dressings with a single function frequently unable to fulfill the complicated and shifting demands in the treatment of diabetic wounds. Some hydrogels exhibit insufficient mechanical robustness, especially when applied to irregularly shaped wounds, which may compromise their adaptability and performance. Additionally, certain hydrogels lack strong bioadhesive properties, ultimately affecting their retention and stability at the location of the wound (Hua et al., 2024; Zeng et al., 2025). Moreover, a major concern with using hydrogels as drug carriers is ensuring the efficient release and bioavailability of the therapeutic agents they hold (Ding et al., 2024). Furthermore, the management of refractory infections during diabetic wound healing continues to pose a substantial clinical challenge. It is therefore essential to develop multifunctional hydrogels that combine effective anti-infective properties with the ability to actively promote wound healing.

Polyethylene glycol (PEG) is advantageous for hydrogel fabrication due to its biocompatibility, water solubility, non-immunogenic nature, adjustable biodegradability, and drug delivery capabilities, as well as its capacity to enhance attachment of cells and proliferation (Wang et al., 2023). Incorporating PEG enhances the physical characteristics of hydrogels, including strength and resilience, improving their suitability for engineering scaffolds for tissues (Ye et al., 2023). Furthermore, PEG serves as an effective drug carrier, enabling sustained and targeted drug release to improve therapeutic outcomes (Li et al., 2022).

Alternatively, catechol-modified quaternary ammonium chitosan exhibits distinct benefits for hydrogel design, such as high biocompatibility, antioxidant activity, rapid gelation rate, and superior mechanical properties. This type of hydrogel not only supports cell encapsulation, but it also forms strong adhesions to inorganic substrates, rendering it an ideal candidate for minimally invasive and targeted cell therapy (Zhu et al., 2018). When combined with sodium bicarbonate and phosphate buffer, thermosensitive injectable hydrogels can be produced, which exhibit enhanced mechanical performances, faster gelation capabilities, and significantly reduced degrees of oxidation. These hydrogels perform exceptionally well in applications requiring high adhesive strength, while also possessing self-healing capabilities, thereby allowing them to maintain appropriate functionality over multiple damage–healing cycles (Zheng et al., 2020). The catechol groups further impart robust antibacterial and antioxidant properties, helping prevent bacterial infection and promoting free-radical scavenging (Wei et al., 2022). Catechol modification enhances adhesion and antioxidant properties while preserving the antimicrobial efficacy of quaternary ammonium compounds, aiding in the prevention and management of chronic diabetic wound infections.

**Figure 1:**
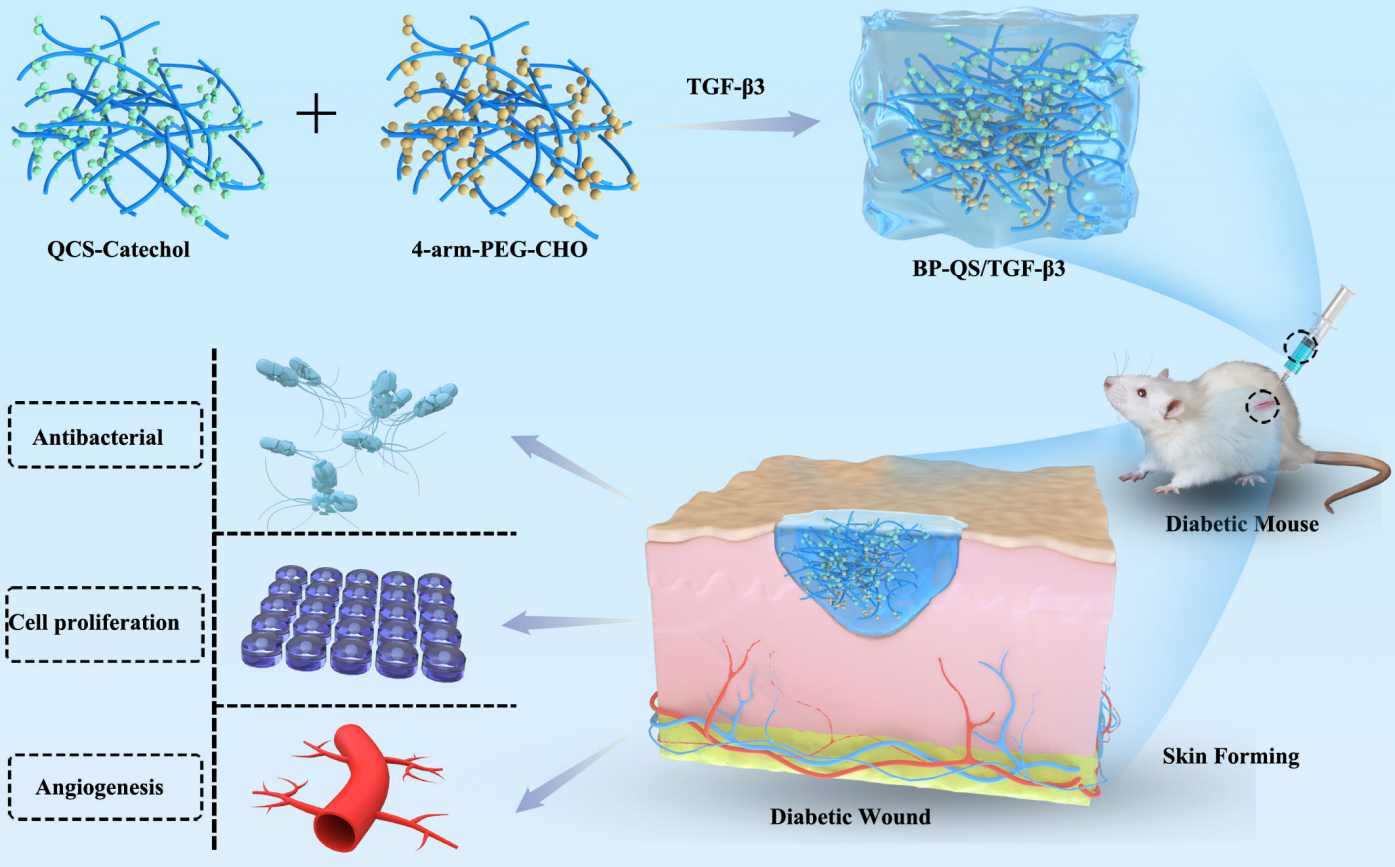
Schematic representation outlining the composite hydrogel and its potential application in diabetic wound healing.

Building upon the respective merits of its components, this study proposes a multifunctional and synergistic therapeutic strategy engineered within a single, injectable hydrogel platform. We hypothesize that the synergistic integration of PEG’s biocompatibility, the robust wet-adhesion and broad-spectrum antibacterial activity of catechol-modified quaternized chitosan, and the sustained release of bioactive TGF-β3 can concurrently disrupt the pathological cycle of diabetic wounds. Unlike conventional dressings that often address only one aspect, our BP-QS/TGF-β3 hydrogel is designed to actively combat biofilm infection, mitigate the hostile wound microenvironment, and directly orchestrate regeneration through enhanced angiogenesis, fibroblast migration, and structured extracellular matrix deposition. This work not only presents a promising candidate for diabetic wound management but also highlights the therapeutic advantage of a unified platform that coordinates multiple healing-promoting events in a spatiotemporally controlled manner, as illustrated in **Figure 1**.

## 2 Materials and methods

### 2.1 Preparation of the BP-QS/TGF-β3 composite hydrogel

Following Zhang et al.’s method (Zhang et al., 2011), 4-arm polyethylene glycol hydroxyl (4-arm PEG-OH) was synthesized by reacting with 4-carboxybenzaldehyde in dichloromethane at 40°C for 24 hours, utilizing dicyclohexylcarbodiimide (DCC) and 4-dimethylaminopyridine (DMAP) as catalysts. The mixture underwent sequential washing with acid, base, and brine, followed by concentration and vacuum drying, yielding benzaldehyde-terminated 4-arm polyethylene glycol (4-arm PEG-CHO) as an off-white solid. Chitosan was dissolved in acetic acid and reacted with trimethylammonium chloride at 60 °C and pH 9–10 for 6–12 h to yield the quaternized chitosan (QCS). An activated catechol solution was combined with QCS dissolved in PBS (pH 5.5–6.0) and allowed to react under nitrogen in darkness for 24 hours, followed by freeze-drying to yield catechol-modified quaternized chitosan (QCS-Catechol). The prepared 4-arm PEG-CHO and QCS-Catechol were dissolved separately in water to give solution concentrations of 7 wt%. The BP-QS hydrogel was formed by combining the two solutions in equal amounts. For the drug-loaded gel BP-QS/TGF-β3, TGF-β3 (1 ng/mL) was initially dissolved in the QCS-Catechol solution before mixing with 4-arm PEG-CHO. The gelation time was ~2–3 min.

### 2.2 Characterization of the composite hydrogel

The structures of the synthesized 4-arm PEG-CHO and QCS-Catechol compounds were confirmed using proton nuclear magnetic resonance (1H NMR) spectroscopy with a Bruker Avance III 400 MHz spectrometer. Fourier transform infrared (FT-IR) spectroscopy was conducted using a Nicolet iS50 spectrometer with KBr pellets. Thirty-two scans were conducted to obtain spectra from 4000 to 400 cm−1 with a 4 cm−1 resolution, analyzing the characteristic vibrational absorption peaks of the hydrogel functional groups. The porous structure of the hydrogel was analyzed using a TESCAN VEGA3 scanning electron microscope (SEM). The freeze-dried hydrogel was fractured in liquid nitrogen, coated with gold, and analyzed at an accelerating voltage of 10 kV.

The swelling ratio of the hydrogel was also evaluated. Specifically, a precisely weighed lyophilized hydrogel sample (5 mm diameter, 2 mm thick) was immersed in PBS (pH 7.4) and allowed to swell in a constant-temperature shaking incubator at 37 °C. At predetermined time intervals, a sample was removed, gently blotted to remove surface moisture, and weighed. The swelling ratio (SR) was calculated as SR = (W_s_ – W_5_)/W_5_ × 100%, where W_s_ is the swollen weight and W_5_ is the initial dry weight. It was considered that swelling equilibrium had been reached when the mass change was <5% over three consecutive measurements.

The hydrogel precursor solution was extruded at room temperature using 18G, 22G, and 26G needles connected to a 1 mL syringe, with a flow rate of 0.5 mL/min. The gelation time and extrusion continuity were subsequently recorded. The adhesive properties were assessed by testing hydrogel attachment to a variety of materials like glass and polystyrene, while its structural stability was evaluated under deformation conditions like bending and twisting. Assessments of tensile capabilities were conducted using a Shimadzu AGS-X universal testing machine, following the ASTM D638 standard. Dumbbell-shaped specimens were elongated at a rate of 10 mm per minute. The Young’s modulus for each specimen was calculated from the gradient of the linear section between 0–10% strain, and the elongation at break was noted as the strain at fracture. Lyophilized hydrogel discs (10 mm diameter, 2 mm thick) were subjected to hydrolytic degradation analysis by immersion in PBS (pH 7.4) and incubation at 37 °C with continuous shaking. Samples were collected at designated intervals, rinsed with PBS, freeze-dried, and weighed to determine the remaining mass percentage. Each experiment was conducted three times.

TGF-β3-loaded hydrogels were incubated in centrifuge tubes with 5 mL of PBS containing 0.1% bovine serum albumin at 37 °C and shaken at 50 rpm for drug release studies. At designated intervals, 1 mL of the release medium was taken out and substituted with the same amount of fresh PBS.TGF-β3 levels were measured with an ELISA kit, and the cumulative release percentage was determined.

### 2.4 *In vitro* biocompatibility evaluation

#### 2.4.1 Experimental groups

L929 mouse fibroblast cells (ATCC^®^ CCL-1™) were maintained at 37 °C in a An environment with 5% carbon dioxide using high levels of glucose DMEM enriched with Serum from fetal bovines at 10%. Group 1 represented the negative control (DMEM with 2% FBS), group 2 was the BP/QS group (100% extract of the BP/QS hydrogel), group 3 was the TGF-β3 group (DMEM with 2% FBS and 1 ng/mL free TGF-β3), and group 4 represented the BP-QS/TGF-β3 group (100% extract of the BP-QS/TGF-β3 hydrogel).

#### 2.4.2 Preparation of the hydrogel extract

The sterilized hydrogel, a diameter of 10 mm and a thickness of 2 mm, was immersed in DMEM with 2% FBS at a 1 cm²/mL ratio and extracted at 37 °C with shaking at 100 rpm for 24 hours. Filtration of the extract was performed using a 0.22 μm membrane and then stored at −80 °C.

#### 2.4.3 CCK-8 cell proliferation assay

L929 cells were planted in 96-well plates with a concentration of 5,000 cells per well. The medium was substituted with the assigned extract or control medium after 24 hours. 10 μL of CCK-8 reagent was added to each well after 24, 48, and 72 hours of incubation, followed by another 2-hour incubation. The absorbance at 450 nm was measured using a microplate reader (Thermo Fisher Scientific, USA). The relative cell viability (%) was determined using the expression:


$$\text{Relative cell viability}(\%)=({\text{OD}}_{\text{experimental}}-{\text{OD}}_{\text{blank}})/({\text{OD}}_{\text{negative control}}-{\text{OD}}_{\text{blank}})\times 100.$$

#### 2.4.4 Analysis of apoptosis by flow cytometry

An Annexin V-FITC/PI apoptosis detection kit was used to stain the gathered L929 cells after 12 hours of treatment, following the instructions provided by the manufacturer. A BD FACSCanto™ II flow cytometer was used to evaluate apoptosis, and FlowJo software was utilized for data analysis.

#### 2.4.5 Hemolysis assay

To evaluate the hydrogel’s hemocompatibility, a hemolysis test was conducted. For this purpose, fresh venous blood from healthy rats was anticoagulated using sodium citrate and diluted with PBS. The diluted blood was incubated with either the test group (100% extract of the BP-QS/TGF-β3 hydrogel), the positive control (deionized water), or the negative control (PBS). Following a 1-hour incubation at 37 °C, the samples underwent centrifugation, and the supernatant’s absorbance was measured at a wavelength of 545 nm using a microplate reader (Thermo Fisher Scientific, USA). Using the equation provided, the hemolysis rate was calculated, and a hemolysis degree below 5% was considered suitable for biomaterial hemocompatibility.


$$\text{Hemolysis}(\%)=({\text{OD}}_{\text{test}}-{\text{OD}}_{\text{negative control}})/({\text{OD}}_{\text{positive control}}-{\text{OD}}_{\text{negative control}})\times 100.$$

#### 2.4.6 Cell migration scratch assay

The L929 cells were introduced into 6-well plates at a rate of 1×10^6 cells per well and allowed to grow until they were completely confluent. A scratch was then made in each well using a pipette tip that holds 200 μL and is sterile. After washing with PBS, the medium was substituted with DMEM that includes 2% FBS and the corresponding treatment. The same location was used to capture images of the scratched area at times of 0, 3, 6, 12, and 18 h (×100 magnification). ImageJ software was used to measure the scratch area, and the cell migration rate along with the wound closure percentage were determined.

#### 2.4.7 *In vitro* antibacterial activity assay

*E. coli* (ATCC 25922) and *S. aureus* (ATCC 6538) were employed as representative Gram-negative and Gram-positive bacterial strains, respectively (purchased from the American Type Culture Collection, USA). Nutrient broth (NB) and nutrient agar (NA) were obtained from Hope Bio-Technology Co., Ltd. (Qingdao, China). Bacterial stocks were activated by streaking onto NA plates and incubating under aerobic conditions at 37 °C for 24 hours. A single colony was inoculated into NB medium and cultured aerobically with shaking at 37 °C until the logarithmic growth phase was reached. Bacterial cells were harvested by centrifugation, resuspended in sterile PBS, and adjusted to a density of ~1×10^8 CFU/mL.

#### 2.4.8 Time-kill assay and determination of antibacterial efficiency

The antibacterial efficacy was quantitatively evaluated using a time-kill assay (Balouiri et al., 2016). Briefly, an aliquot (100 μL of the homogenized BP-QS/TGF-β3 hydrogel precursor solution) or an equal volume of sterile PBS (negative control) was added to 10 mL of NB containing 1% bacterial inoculum (final concentration ~10^6 CFU/mL). The cultures were incubated at 37 °C with shaking at 180 rpm for 18 hours. Subsequently, 100 μL of the bacterial suspension from each group was subjected to a series of ten-fold serial dilutions (10^-1 to 10^-6) in sterile PBS. The number of viable bacteria was then determined using the plate counting method: 100 μL of the appropriate dilutions were spread onto NA plates and incubated aerobically at 37 °C for 24 hours. Colonies were manually counted on plates containing between 30 and 300 colonies. The antibacterial efficiency was calculated as follows (Balouiri et al., 2016):


Antibacterial efficiency(%)=[(CFU_control–CFU_test)/CFU_control]×100

where CFU_control and CFU_test represent the number of colony-forming units per milliliter from the control and test groups, respectively.

#### 2.4.9 Agar diffusion assay

For the agar diffusion assay, the adjusted bacterial suspension was mixed with molten NA medium (cooled to ~50 °C) to achieve a final concentration of approximately 1×10^6 CFU/mL in the agar layer. The mixture was poured onto plates to form a uniform bacterial lawn. After solidification, wells (6 mm diameter) were punched, and an aliquot (50 μL) of the homogenized BP-QS/TGF-β3 hydrogel precursor solution, PBS (negative control), or ampicillin solution (1 mg/mL, positive control) was added. The diameter of the inhibition zone (including the well) was measured with a digital caliper after aerobic incubation at 37 °C for 24 hours.

### 2.5 Analysis of wound healing promotion by BP-QS/TGF-β3 in diabetic mice

#### 2.5.1 Establishment of an animal model and evaluation of the wound repair capacity

The Animal Ethics and Welfare Committee of the 900th Hospital of the Joint Logistics Support Force reviewed and approved all animal experiments conducted in this study. (Approval No.2024-33). All activities strictly followed the ‘3R Principles’ for animal welfare and complied with the NIH Guide for the Care and Use of Laboratory Animals.

Male C57BL/6J mice aged eight weeks, with a weight range of 20–22 grams, were utilized under conditions that are free of particular pathogens. After a two-week period of getting used to the environment, the mice were exposed to a 12-hour fast with access to water. The diabetic model was induced by injecting streptozotocin (STZ, 50 mg/kg) into the peritoneum daily for a period of five days. Diabetes was verified when fasting blood glucose levels consistently reached or surpassed 16.7 mmol/L.

Isoflurane was used to anesthetize the mice, their dorsal hair was shaved off, and depilatory cream was applied to clean their skin. The skin was disinfected with iodophor, and a full-thickness skin defect wounds, each 1 mm in diameter and extending to the fascia layer using a sterile biopsy punch. Immediately after wound creation, photographic images were recorded (Day 0).Diabetic mice were randomly assigned to four groups (n = 6): the PBS group (topically applied PBS), the BP-QS group (topically applied BP/QS hydrogel), the TGF-β3 group (injected with free TGF-β3 solution), and the BP-QS/TGF-β3 group (topically applied BP-QS/TGF-β3 hydrogel).Dressings were replaced bi-daily, with wound images taken on days 0, 2, 4, 8, and 12.The wound area was quantified using ImageJ software, and the relative wound healing ratio was determined accordingly (Xue et al., 2025).


Relative wound deficit ratio(%)=(Current area/Initial area)×100

### 2.6 Histological analysis and systemic toxicity assessments

Wound tissues were harvested on days 2, 8, and 12, fixed in 4% paraformaldehyde, and embedded in paraffin for sectioning. Hematoxylin and eosin (H&E) staining, Masson’s trichrome staining, and immunohistochemical staining (CD31, Ki67) were performed. The vascular density, cell proliferation index, and collagen deposition were quantitatively analyzed using ImageJ software. After performing these experiments, the primary organs, including the heart, liver, spleen, lungs, and kidneys, were gathered for H&E staining. Histopathological evaluations were performed by a blinded pathologist to assess for potential tissue damage.

### 2.7 Statistical analysis

All experimental results are presented as the average ± standard deviation (average ± SD). The statistical analysis performed using GraphPad Prism 8.0. One-way analysis of variance (ANOVA) was used to compare the groups. If the assumption of homogeneity of variance was satisfied, Tukey’s multiple comparisons test was applied for *post hoc* analysis; otherwise, an appropriate non-parametric test was used. The statistical analysis for experiments involving only two groups was performed using Student’s t-test. A significance level of *p* < 0.05 was adopted, with *p* < 0.05 indicating a significant difference, *p* < 0.01 indicating a highly significant difference, and *p* < 0.001 indicating an extremely significant difference.

## 3 Results

### 3.1 Morphology, swelling properties, adhesion, twisting, bending, and detachment behavior of the hydrogel

#### 3.1.1 Macroscopic and microscopic morphologies

**Figure 2:**
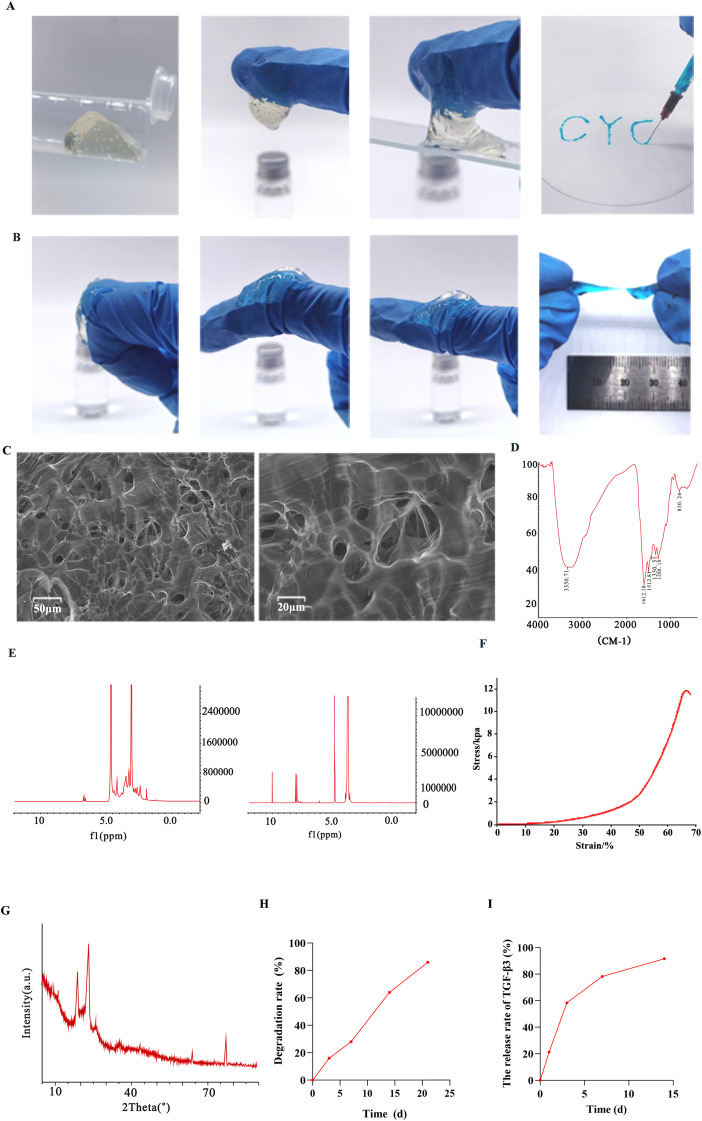
Characterization of the BP-QS/TGF-β3 hydrogel. **(A)** (1) The light-yellow lyophilized BP-QS/TGF-β3 powder. (2) The transparent hydrated hydrogel, which forms a homogeneous, bubble-free elastic solid after gelation. (3) Adhesion of the hydrogel to a glass surface. (4) Injectability of the hydrogel. **(B)** (1-3) Flexibility of the hydrogel under bending deformation at 90°, 45°, and 0°. (4) Tensile fracture strain of the hydrogel. **(C)** Porous structure of the hydrogel, showing an irregular pore morphology with various sizes. **(D)** Fourier Transform Infrared (FT-IR). **(E)** NMR Characterization of Precursors:(1) 4-arm PEG-CHO; (2) QCS-Catechol. **(F)** Stress–Strain Curve. **(G)** X-ray Diffraction. **(H)** Triphasic degradation behavior of the BP-QS/TGF-β3 hydrogel: A rapid degradation phase (0–7 d) with 28.4% ± 2.1% degradation; a sustained degradation phase (7–14 d) with 55.7% ± 1.8% degradation; and an accelerated degradation phase (14–21 d) with 82.3% ± 2.5% degradation. **(I)** TGF-β3 release profile showing typical biphasic characteristics: Cumulative release of 19.83% ± 3.4% within 12 h, 58.4% ± 2.7% on day 3, 78.2% ± 2.9% on day 7, and 91.5% ± 3.3% on day 14.

The lyophilized BP-QS/TGF-β3 powder appeared light yellow in color, while the hydrated hydrogel was transparent and formed a homogeneous, bubble-free elastic solid after gelation (**Figures 2A-1, A-2**). SEM imaging (**Figure 2C**) revealed a porous microstructure with an irregular pore morphology and size distribution. The pore diameters ranged from several micrometers (e.g., 5–10 μm) to ~30 μm, reflecting the heterogeneous nature of the pore architecture. The pore walls and gel matrix exhibited continuity and flexibility, contributing to an overall loose and interconnected structure.

#### 3.1.2 Injectability and gelation kinetics

The hydrogel demonstrated favorable injectability (**Figures 2A**, **4**), extruding smoothly without clogging through a needle attached to a 1 mL syringe, indicating its suitability for clinical applications. Gelation occurred within 2–3 min at room temperature. No liquid dripping was observed at the needle tip during extrusion, and the injected hydrogel formed continuous blue gel strands in an aqueous medium without dissolution or floating, confirming rapid gel formation. The extruded hydrogel strands (1–2 mm diameter) remained intact without fracture or dissolution in PBS.

#### 3.1.3 Adhesion performance, flexibility, deformation behavior and swelling properties

**Figure 3:**
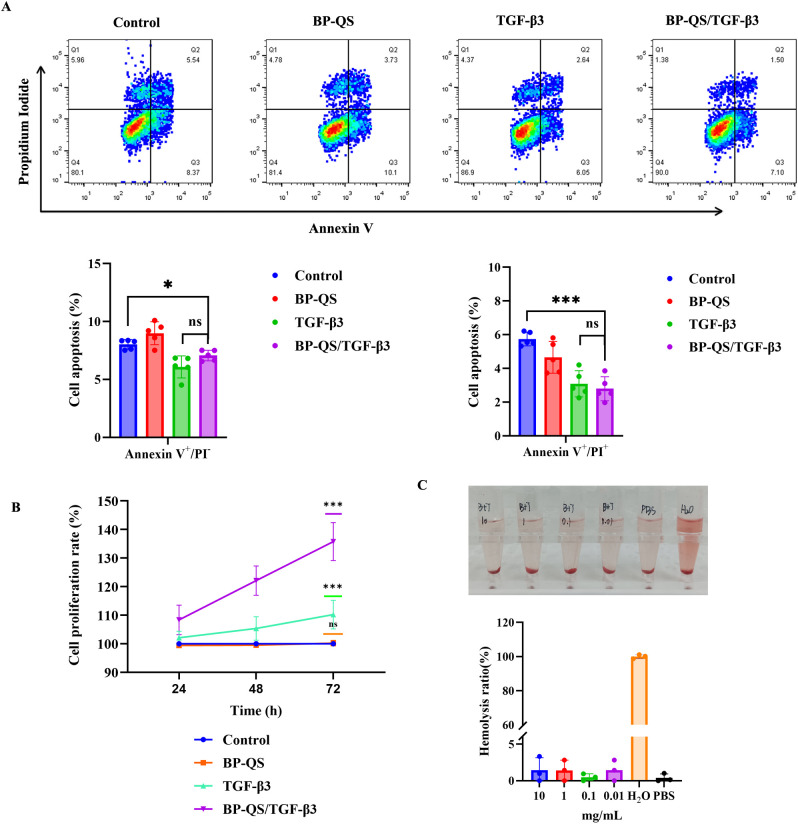
Biocompatibility evaluations. **(A)** Both the TGF-β3 and BP-QS/TGF-β3 groups significantly reduce the rates of early apoptosis (*p* < 0.05) and late apoptosis/necrosis (*p* < 0.001). The relative proliferation rate of the BP-QS/TGF-β3 group is significantly higher than that of the TGF-β3 group at both 48 and 72 h (*p* < 0.05). **(B)** CCK-8 assay results, revealing that after 72 h of culture, the relative cell viability in the BP-QS group is 98.7% ± 3.5% (*p* > 0.05, c.f., the negative control), indicating no significant cytotoxicity for the material itself. In contrast, the BP-QS/TGF-β3 group shows significantly enhanced cell proliferation, with a viability of 135.2% ± 6.8% (*p* < 0.001 vs. the control group). **(C)** Hemolysis assay results, indicating <5% hemolysis for BP-QS/TGF-β3 and demonstrating no significant hemolytic toxicity. *p < 0.05, **p < 0.01, ***p < 0.001; ns, p > 0.05.

The adhesive strength of the hydrogel was subsequently evaluated (**Figures 2A**, **3**). The 90° peel force on glass measured 5.2 ± 0.3 N, with an adhesion energy of 28.5 ± 2.1 J/m^2^, demonstrating strong adhesion characteristics and indicating the suitability of this material for use in dynamic tissues, such as wounds. In the twist tests, the hydrogel strips (50 mm × 5 mm) withstood 10 cycles of 180° twisting without cracking. Meanwhile, the bending tests showed a minimum bending radius of 5 mm (comparable to finger joint flexion), with no structural failure being observed after repeated bending (**Figure 2B**). The equilibrium swelling ratio of the stabilized BP-QS/TGF-β3 hydrogel was determined to be 116.7%.

### 3.2 Mechanical properties: stress–strain curve, young’s modulus, and elongation at break

**Figure 4:**
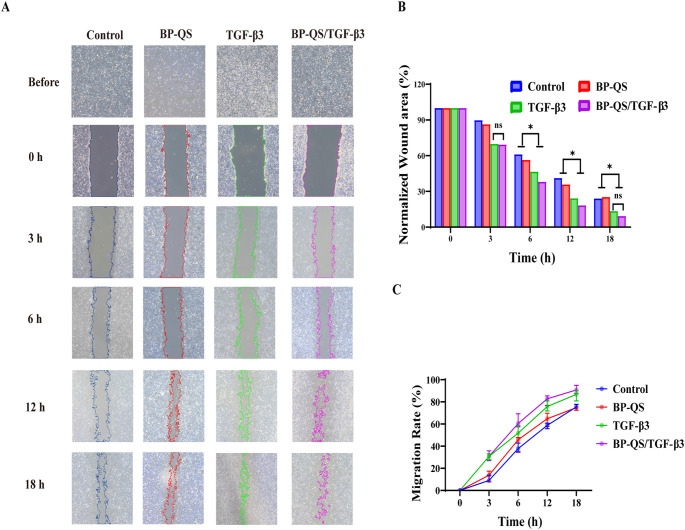
Cell scratch assay. **(A)** Representative microscopic images of cell scratch wound healing in each group. **(B)** Quantitative analysis of the wound closure area ratio. No significant difference is observed between the BP/QS group and the control group (*p* > 0.05), indicating that the hydrogel material itself has no significant effect on cell migration. Compared with the control group, the BP-QS/TGF-β3 group shows the most significant promotion of cell migration, while the TGF-β3 alone group also exhibits a certain facilitatory effect. **(C)** Cell migration analysis. Both the TGF-β3 and BP-QS/TGF-β3 groups demonstrate significantly faster migration rates than the control group at all time points (*p* < 0.05), confirming their promotive effects on fibroblast migration, with the most notable advantage being observed at 6 and 12 h. *p < 0.05, **p < 0.01, ***p < 0.001; ns, p > 0.05.

As shown in **Figure 2F**, the BP-QS/TGF-β3 hydrogel exhibited typical elastomeric mechanical behavior. Specifically, in the initial linear region (0–10% strain), the stress increased linearly with the strain, reflecting the elastic response of the material under small deformations. A yield point occurred at ~50% strain, beyond which the crosslinked network began to rupture partially, resulting in a reduced rate of stress increase. Fracture occurred at a strain of 264.3% ± 12.5%, corresponding to a tensile strength of 15.3 ± 1.2 kPa. The Young’s modulus, calculated from the slope of the linear region, was 10.2 ± 0.8 kPa, while the elongation at break reached 264.3% ± 12.5% (**Figures 2B**, **4**).

### 3.3 X-ray diffractometry and fourier transform infrared spectroscopy

X-ray diffractometry (XRD, **Figure 2G**) revealed a significant reduction in the intensity of the characteristic PEG diffraction peaks for the BP-QS/TGF-β3 composite hydrogel at 19.2° (110 plane) and 23.4° (112 plane), indicating that the PEG segments exhibited decreased crystallinity due to crosslinking with the QCS-Catechol component. Broad amorphous halos appeared at 10.5° and 20.1°, corresponding to the amorphous structure of chitosan. The full width at half maximum of the PEG crystalline peak at 19.2° increased from 0.18° to 0.35°, further confirming that molecular chain entanglement inhibited the regular arrangement of the PEG structure.

FT-IR spectroscopy confirmed the successful synthesis of the target QCS-Catechol and 4-arm PEG-CHO structures, as well as the formation of a chemically crosslinked composite hydrogel without detectable impurities (**Figure 2D**).

### 3.4 NMR Characterization of the precursors

#### 3.4.1 ^1^H NMR analysis of 4-arm PEG-CHO

The ^1^H NMR spectrum presented in **Figure 2E-1** clearly shows numerous characteristic signals, including a distinct singlet at δ 9.98 ppm (corresponding to the formyl proton) and aromatic proton signals at δ 7.82 ppm (d, *ortho* protons) and δ 7.48 ppm (t, *meta* protons), confirming successful grafting of benzaldehyde. The strong peak at δ 3.64 ppm was attributed to the –CH_2_CH_2_O– repeating units of the PEG backbone.

#### 3.4.2 ^1^H NMR analysis of QCS-catechol

The ^1^H NMR spectrum presented in **Figure 2E-2** displays a sharp singlet at δ 3.28 ppm, corresponding to the protons of the quaternary ammonium group (–N^+^(CH_3_)_3_), along with multiplets in the range of δ 6.72–6.88 ppm, which correspond to the aromatic protons of the catechol moiety. Additionally, characteristic signals corresponding to the chitosan backbone were detected at δ 4.98 ppm (d, anomeric C1-H), δ 3.56–3.92 ppm (m, C3–C6-H), and δ 2.86 ppm (t, C2-H).

### 3.5 *In vitro* degradation kinetics and TGF-β3 release profile of the hydrogel

The BP-QS/TGF-β3 hydrogel exhibited triphasic degradation behavior in PBS (pH 7.4, 37 °C, as shown in **Figure 2H**. Specifically, in the rapid degradation phase (0–7 d), 28.4% ± 2.1% degradation was observed, which was primarily caused by hydrolysis of the surface PEG segments. In the subsequent sustained degradation phase (7–14 d), the degradation rate slowed, with a cumulative mass loss of 55.7% ± 1.8% by day 14. During the accelerated degradation phase (14–21 d), 82.3% ± 2.5% degradation was observed due to enzymatic cleavage of the chitosan backbone.

The release profile of TGF-β3 from the BP-QS/TGF-β3 hydrogel showed typical biphasic kinetics (**Figure 2I**), which began with an initial burst release phase (21.3% ± 2.4% within the first 12 h) and was followed by a cumulative release phase (reaching 19.83% ± 3.4% at 12 h and 58.4% ± 2.7% on day 3). In the final sustained release phase, the cumulative release reached 78.2% ± 2.9% on day 7 and 91.5% ± 3.3% on day 14, indicating near-complete release.

### 3.6 *In vitro* biocompatibility analysis

#### 3.6.1 Flow cytometric assessment of the hydrogel cytotoxicity

The effects of different treatments on L929 cell apoptosis and necrosis were evaluated using Annexin V-FITC/PI double staining, as shown in **Figure 3A**. Compared with the control group, the BP-QS hydrogel alone (BP/QS group) did not induce significant increases in the early apoptosis or late apoptosis/necrosis rates (*p* > 0.05). In contrast, treatments containing TGF-β3 (TGF-β3 group and BP-QS/TGF-β3 group) exhibited significant beneficial effects, wherein both significantly reduced the rates of early apoptosis (*p < 0.05) and late apoptosis/necrosis (***p < 0.001).

#### 3.6.2 CCK-8 cell proliferation assay

As shown in **Figure 3B**, the CCK-8 assay results demonstrate that after 72 h of culture, the relative cell viability in the BP-QS group was 98.7% ± 3.5% (*p* > 0.05 vs. control), indicating that the prepared material exhibited no intrinsic cytotoxicity. In the BP-QS/TGF-β3 group, cell proliferation was significantly enhanced, with a viability of 135.2% ± 6.8% (vs. control, *p* < 0.001) being recorded at 72 h.

#### 3.6.3 Hemolysis test

As shown in **Figure 3C**, hemolysis tests demonstrated that the extracts of BP-QS/TGF-β3 at various concentrations (0.01, 0.1, 1, and 10 mg/mL) all exhibited hemolysis rates below 5%, suggesting the absence of significant hemolytic toxicity.

#### 3.6.4 Cell migration assessment using the scratch assay

A scratch assay was performed to evaluate the effects of different treatments on the migration ability of L929 cells. As shown in **Figure 4**, the BP/QS group showed no significant difference compared with the control group (*p* > 0.05), indicating that the hydrogel material itself did not significantly affect cell migration. In contrast, both the TGF-β3 and BP-QS/TGF-β3 groups demonstrated significantly enhanced cell migration compared with the control (*p* < 0.05). The BP-QS/TGF-β3 group exhibited the most pronounced promotive effect, with notably faster wound closure being observed, particularly at the earlier time points of 6 and 12 h.

### 3.7 Analysis of the *in vitro* antibacterial performance

**Figure 5:**
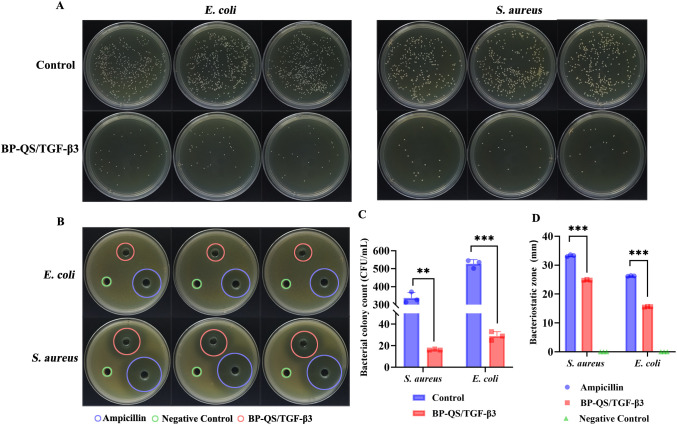
*In vitro* antibacterial assay. **(A)** Antibacterial activity test, **(B)** inhibition zones, and **(C)** statistical analysis of the antibacterial efficiency. Compared with the control group, treatment with the BP-QS/TGF-β3 hydrogel precursor solution significantly reduces the number of viable bacteria (*S. aureus*: *p* < 0.01; *E*. *coli*: *p* < 0.001). The antibacterial efficiencies against *S. aureus* and *E*. *coli* reach 95.3% ± 0.8% and 94.6% ± 1.2%, respectively. **(D)** Statistical analysis of the inhibition zone diameter. The BP-QS/TGF-β3 hydrogel precursor solution forms distinct inhibition zones against both *S. aureus* and *E*. *coli*, with diameters of 24.89 ± 0.17 and 15.62 ± 0.11 mm, respectively, showing a statistically significant difference compared with the negative control group (*p* < 0.001). *p < 0.05, **p < 0.01, ***p < 0.001; ns, p > 0.05.

The antibacterial properties of the BP-QS/TGF-β3 hydrogel precursor solution against *S. aureus* and *E. coli* were assessed via the plate counting technique. As shown in **Figure 5**, treatment with the BP-QS/TGF-β3 hydrogel precursor solution notably decreased the viable bacteria count for both strains compared to the control group (*p* < 0.01). The hydrogel demonstrated strong antibacterial efficacy, achieving inhibition efficiencies of 95.3% ± 0.8% for *S. aureus* and 94.6% ± 1.2% for *E. coli*.

The agar diffusion assay further confirmed the diffusion-based antibacterial activity of the BP-QS/TGF-β3 hydrogel precursor solution. Clear inhibition zones were observed for both *S. aureus* and *E. coli*, with diameters of 24.89 ± 0.17 and 15.62 ± 0.11 mm, respectively. Although these values were smaller than those of the ampicillin group (i.e., the positive control), the BP-QS/TGF-β3 hydrogel precursor solution still exhibited considerable antibacterial diffusion ability. A statistically significant difference (*p* < 0.001) was observed compared with the negative PBS control, which produced no inhibition zone.

### 3.8 Evaluation of the BP-QS/TGF-β3 hydrogel in promoting diabetic wound healing

#### 3.8.1 Animal model establishment and validation

**Figure 6:**
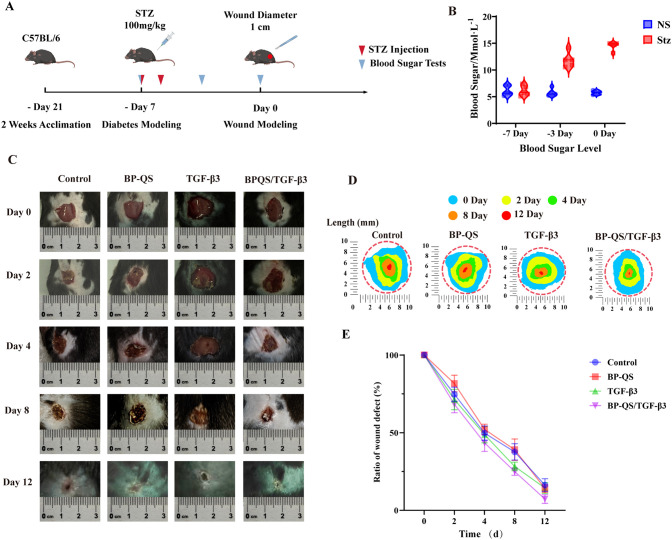
Macroscopic progression and quantitative analysis of wound healing in diabetic mice. **(A)** Schematic diagram outlining the establishment of a diabetic mouse wound model. **(B)** Blood glucose monitoring in diabetic mice. **(C)** Macroscopic progression of wound healing in diabetic mice, wherein the BP-QS/TGF-β3 group exhibits the fastest healing rate, with substantial granulation tissue formation being observed on day 4. By day 8, the wound is largely covered with new epithelium, and almost complete closure is achieved by day 12. **(D)** Thermal maps illustrating wound healing in each group. **(E)** Quantitative analysis of the relative wound deficit ratio in diabetic mice, wherein the BP-QS/TGF-β3 composite hydrogel demonstrates an outstanding pro-healing efficacy. On day 2, the relative wound deficit ratio in the BP-QS/TGF-β3 group (68.24% ± 5.41%) is significantly lower than that of the control group (74.72% ± 4.47%, *p* < 0.05). By day 4, the deficit ratio (41.20% ± 5.92%) is significantly lower than those of the TGF-β3 group (49.88% ± 5.37%, *p* < 0.05) and the BP-QS group (50.18% ± 3.42%, *p* < 0.05). On day 8, the advantage of the BP-QS/TGF-β3 group becomes more pronounced, with a deficit ratio (24.90% ± 2.69%) that is significantly lower than those of the TGF-β3 group (30.26% ± 4.21%, *p* < 0.05) and the BP-QS group (38.36% ± 7.26%, *p* < 0.01). By day 12, the wounds in the BP-QS/TGF-β3 group are nearly completely healed, with the deficit ratio decreasing to the lowest value (7.30% ± 2.76%), which is significantly superior to those of the TGF-β3 group (15.82% ± 3.42%, *p* < 0.01) and the BP-QS group (13.33% ± 2.89%, *p* < 0.01).

A diabetic mouse model was successfully established using STZ induction, with a modeling success rate of 85%. As shown in **Figures 6A, B**, demonstrate a significant increase in blood glucose levels in all diabetic groups compared to the control group (p < 0.01). The groups showed no significant differences in initial blood glucose levels or body weights *(p* > 0.05), indicating consistent baseline conditions. A 10 mm full-thickness skin defect was created on the dorsal side of each mouse, with initial wound areas showing no significant differences between groups, thus satisfying the experimental criteria.

#### 3.8.2 Macroscopic healing process and quantitative analysis

Although all wounds showed progressive healing over time, the healing rates differed markedly among the different treatment groups (**Figures 6C, D**). In the control group, the wounds healed slowly and remained open on day 12. The BP-QS group exhibited faster healing than the control; however, complete closure was not achieved by day 12. In contrast, the TGF-β3 group demonstrated significantly accelerated wound healing with robust granulation tissue formation. The BP-QS/TGF-β3 group showed the most rapid healing, wherein substantial granulation tissue appeared by day 4, the wound was largely covered by new epithelium by day 8, and almost complete closure was observed by day 12.

Quantitative analysis of the relative wound deficit ratio clearly indicated the superior pro-healing efficacy of the BP-QS/TGF-β3 hydrogel (**Figure 6E**). On day 2, the BP-QS/TGF-β3 group already showed a significantly lower deficit ratio (68.24% ± 5.41%) compared with that of the control (74.72% ± 4.47%, *p* < 0.05). By day 4, the deficit ratio in the BP-QS/TGF-β3 group (41.20% ± 5.92%) was significantly lower than those in both the TGF-β3 (49.88% ± 5.37%, *p* < 0.05) and BP-QS (50.18% ± 3.42%, *p* < 0.05) groups. This advantage became more pronounced on day 8, with a deficit ratio of 24.90 ± 2.69% being recorded for the BP-QS/TGF-β3 group, which is significantly lower than those in the TGF-β3 (30.26% ± 4.21%, *p* < 0.05) and BP-QS (38.36% ± 7.26%, *p* < 0.01) groups. By day 12, the BP-QS/TGF-β3 group reached the lowest deficit ratio (7.30% ± 2.76%), significantly outperforming both the TGF-β3 (15.82% ± 3.42%, *p* < 0.01) and BP-QS (13.33% ± 2.89%, *p* < 0.01) groups. These results demonstrate that the BP-QS/TGF-β3 hydrogel sustainably accelerates diabetic wound healing and exhibits significant pro-healing effects throughout the entire process.

#### 3.8.3 Histological analysis of the wound tissues

**Figure 7:**
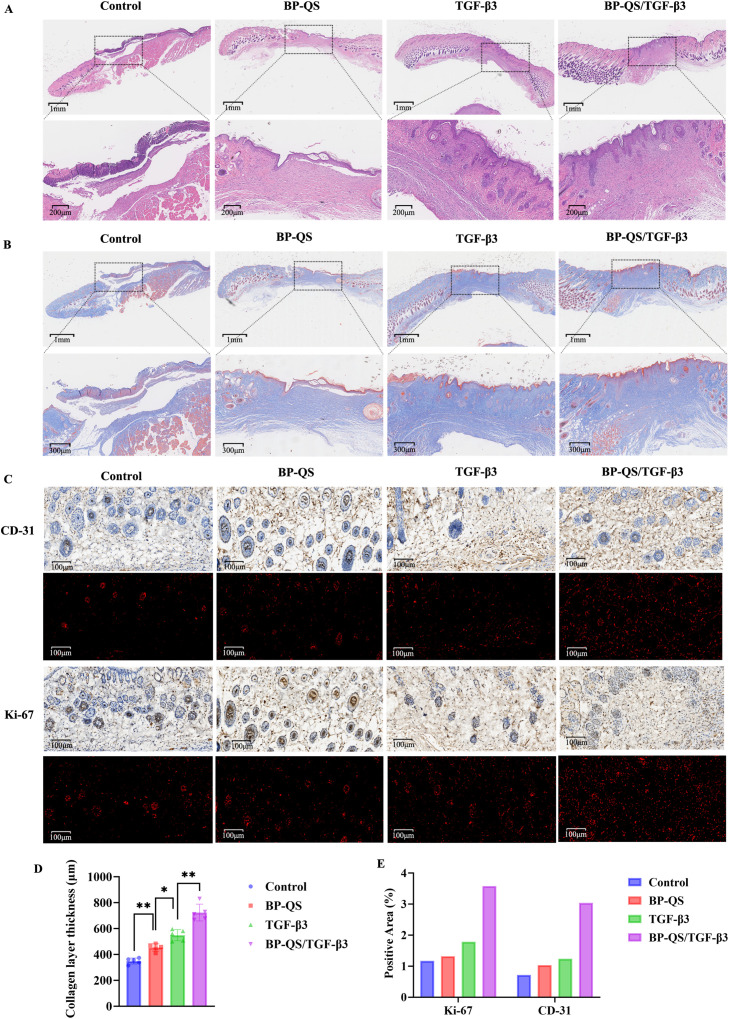
Histological analysis of the wound tissues. **(A)** H&E staining of the wound sections at (1) 10× magnification and (2) 50× magnification. **(B)** Masson’s trichrome staining of the wound sections at (1) 10× magnification, (2) 40× magnification. **(C)** Immunohistochemical investigations: (1) CD31 expression, (2) fluorescence distribution of CD31, (3) Ki-67 expression, and (4) fluorescence distribution of Ki-67. **(D)** Statistical analysis of the collagen area based on Masson’s trichrome staining, wherein the BP-QS/TGF-β3 composite hydrogel group exhibits the most significant promotion of collagen deposition. The thickness of the fibrous layer in this group is 724.61 ± 60.12 μm, which is significantly greater than those in the TGF-β3 group (549.28 ± 41.97 μm, *p* < 0.01), the BP-QS group (454.64 ± 26.32 μm, *p* < 0.001), and the control group (350.00 ± 21.62 μm, *p* < 0.001). **(E)** Comparison of the CD31- and Ki-67-positive proportions. The BP-QS/TGF-β3 group exhibits significantly higher positive expression levels of Ki-67 (3.578%) and CD31 (3.039%) in the wound tissues compared with those exhibited by the other groups. *p < 0.05, **p < 0.01, ***p < 0.001; ns, p > 0.05.

Histological evaluations performed using H&E and Masson’s trichrome staining on day 12 post-wounding revealed distinct differences among the groups (**Figures 7A, B**). Compared with the control group, which exhibited disordered and fragile subcutaneous structures, the BP-QS, TGF-β3, and BP-QS/TGF-β3 groups all exhibited improved wound healing at the skin–wound junction, with more substantial and organized subcutaneous tissue being evident. Among these, the TGF-β3 and BP-QS/TGF-β3 groups displayed denser fibrous connections within the subcutaneous tissue. In particular, the BP-QS/TGF-β3 group demonstrated the most robust wound repair, characterized by a thick fibrous layer, highly ordered tissue architecture, and elevated matrix density.

Masson’s trichrome staining further indicated that, compared with the other groups, the BP-QS/TGF-β3 group exhibited the most orderly arrangement and highest density of collagen fibers, accompanied by earlier maturation compared (**Figure 7B**). Quantitative analysis of the collagen layer thickness based on the Masson’s staining results confirmed these observations (**Figure 7D**). Specifically, the BP-QS/TGF-β3 hydrogel group showed the most pronounced enhancement in collagen deposition, with a fibrous layer thickness of 724.61 ± 60.12 μm, which is significantly greater than those observed for the TGF-β3 group (549.28 ± 41.97 μm, *p* < 0.01), the BP-QS group (454.64 ± 26.32 μm, *p* < 0.001), and the control group (350.00 ± 21.62 μm, *p* < 0.001). These results indicate that the BP-QS/TGF-β3 hydrogel effectively promotes collagen synthesis and deposition, thereby accelerating tissue repair and maturation.

Immunohistochemical quantitative analysis further supported these findings (**Figures 7C, E**). In this case, the BP-QS/TGF-β3 group exhibited significantly higher proportions of Ki-67-positive cells (3.578%) and CD31-positive cells (3.039%) in the wound tissue compared to those detected for the other groups. Specifically, the cell proliferation activity (Ki-67) in this group was 3.05-, 2.71-, and 2.00-times higher than those of the control, BP-QS, and TGF-β3 groups, respectively. Similarly, the angiogenesis level (CD31) was 4.22-, 2.93-, and 2.45-times higher, respectively. These results demonstrate that the BP-QS/TGF-β3 hydrogel significantly enhances both cell proliferation and vascularization during wound healing.

#### 3.8.4 Systemic toxicity analysis

**Figure 8:**
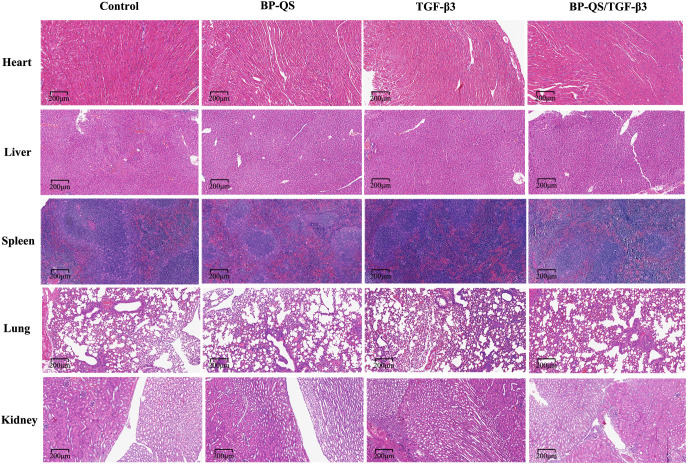
Systemic toxicity analysis of the major organs in mice. Histological analysis of the major organs (heart, liver, spleen, lungs, and kidneys) showing no obvious pathological changes (e.g., necrosis, inflammatory infiltration, fibrosis, or structural abnormalities) in any of the experimental groups.

As shown in **Figure 8**, histological examination of the major organs (heart, liver, spleen, lungs, and kidneys) revealed no significant pathological alterations (e.g., necrosis, inflammatory infiltration, fibrosis, or structural abnormalities) in any of the groups. No notable differences were observed between the BP-QS/TGF-β3 group and the control or other experimental groups, indicating that the hydrogel did not induce systemic toxicity under *in vivo* conditions and possesses a favorable biosafety profile.

## 4 Discussion

Chitosan, a natural polysaccharide, is an excellent choice for wound dressings because of its compatibility with biological tissues, ability to break down naturally, and antibacterial characteristics (Matica et al., 2019). The study showed that the BP-QS/TGF-β3 hydrogel effectively inhibits *S. aureus* and *E. coli* (inhibition efficiency >94%) due to the antibacterial properties of quaternized chitosan and the synergistic effects of catechol groups. Furthermore, the hydrogel showed favorable hemocompatibility and cytocompatibility, with no significant cytotoxicity being observed. Histopathological examination of the major organs from the treated mice revealed no pathological changes, indicating a good *in vivo* biosafety profile and confirming the potential clinical application of the prepared hydrogel.

Over the past few years, chitosan-based hydrogels have attracted extensive research interest due to their remarkable efficacy in promoting wound healing. Studies show they contribute to the healing of wounds associated with diabetes by adjusting the wound microenvironment and promoting angiogenesis, cell proliferation, and collagen deposition (Li, 2025). Indeed, the multifunctionality of chitosan-based hydrogels offers significant advantages in the treatment of diabetic wounds. They facilitate wound healing by shifting macrophages to the M2 phenotype, which reduces inflammation and promotes tissue regeneration (Wei et al., 2024). Additionally, chitosan-based hydrogels can alleviate oxidative stress and hypoxic conditions by scavenging excess reactive oxygen species and supplying oxygen, thereby improving the wound-healing environment (Wang et al., 2025). These properties highlight the great application potential of chitosan-based hydrogels as diabetic wound dressings.

Chitosan-based hydrogels are distinguished by their self-healing capability, making them highly effective for treating complex and irregularly shaped wounds. Through dynamic Schiff base reactions, the structural integrity of chitosan-based hydrogels can be rapidly restored after mechanical damage, thereby enhancing their durability in practical applications (Pan et al., 2022; Li et al., 2023a). Incorporating bioactive components like silver nanoparticles and polyphenolic compounds can enhance the antibacterial and antioxidant properties of chitosan-based hydrogels, providing comprehensive protection for diabetic wounds (Masood et al., 2019; Zhao et al., 2023).

TGF-β3 is increasingly recognized for its crucial role in diabetic wound repair, as it modulates inflammation and enhances tissue regeneration. Specifically, by regulating the inflammatory microenvironment, TGF-β3 can facilitate the wound healing process. Indeed, one study reported that TGF-β3 is highly expressed during the inflammatory phase of ischemic ulcers, with reduced activity in subsequent phases, thereby suggesting its stage-specific function in wound repair (Biniazan et al., 2022). Furthermore, the involvement of TGF-β3 in diabetic wound healing has been further validated. Research indicates that TGF-β3 improves the healing of wounds by modulating matrix metalloproteinase-9 (MMP-9) expression. In this case, the combination of *p*-coumaric acid with a collagen scaffold has been shown to enhance TGF-β3 expression while reducing MMP-9 levels, ultimately shortening the inflammatory phase and enhancing both angiogenesis and tissue regeneration (Selvakumar and Lonchin, 2023). The results underscore TGF-β3’s potential as a therapeutic agent in managing diabetic wounds. TGF-β3 has been shown to interact with additional signaling pathways to enhance wound healing. Exosomes from adipose stem cells facilitate scarless healing and mitigate fibrosis via the miR-204-5p/TGF-β1/Smad pathway (Song et al., 2025), suggesting that TGF-β3 synergizes with other growth factors and signaling molecules to markedly improve diabetic wound healing. In the nervous system, TGF-β3 derived from de-differentiated Schwann cells is crucial for promoting wound healing. Studies have revealed that TGF-β3 facilitates the migration of fibroblasts and keratinocytes, thereby accelerating the closure of chronic wounds (Ou et al., 2022).

This evidence highlights the critical function of TGF-β3 in diabetic wound healing. Leveraging the inherent advantages of chitosan and TGF-β3, the BP-QS/TGF-β3 hydrogel demonstrates excellent injectability, rapid gelation (within 2–3 min), and strong tissue adhesion (adhesion energy = 28.5 ± 2.1 J/m^2^). These properties significantly enhance its suitability for clinical application and its stability in dynamic wound environments. Moreover, the high fracture elongation (264.3% ± 12.5%) and suitable Young’s modulus (10.2 ± 0.8 kPa) exhibited by this hydrogel indicate that it possesses desirable flexibility and a suitable mechanical strength, ultimately enabling it to accommodate wound contraction and body movement, while effectively protecting the wound site. XRD and NMR analyses verified the synthesis of 4-arm PEG-CHO and QCS-Catechol, along with their successful cross-linking in the composite hydrogel. Additionally, *in vitro* degradation tests revealed a triphasic degradation behavior, consistent with the hydrolysis of PEG segments and the enzymatic cleavage of chitosan. This controlled degradation profile supports the sustained release of TGF-β3, providing a foundation for its prolonged biological activity. Furthermore, the experimental design for evaluating the antibacterial activity warrants clarification. The potent antibacterial efficacy observed against both S. aureus and E. coli is attributed to the intrinsic properties of the hydrogel’s structural components, namely the quaternized chitosan and catechol groups. It is important to note that the antibacterial assays were conducted using the final, integrated BP-QS/TGF-β3 formulation. This approach was chosen because the primary objective was to validate the performance of the complete therapeutic system, which is designed to concurrently address infection and promote healing in the complex diabetic wound microenvironment. Given that the cytokine TGF-β3 itself possesses no known direct antibacterial function, the inclusion of a TGF-β3-only control was deemed unnecessary for this specific assessment. Conversely, the biocompatibility and cell migration assays employed a more decomposed experimental design (including blank hydrogel and free TGF-β3 groups) to precisely delineate the contribution of each component—the biocompatible scaffold and the bioactive factor—to the overall pro-healing effect. This stratified experimental strategy allows for a more targeted and efficient validation of the multifunctional hydrogel’s key attributes.

The release kinetics of TGF-β3 displayed a biphasic pattern, characterized by an initial burst for rapid healing initiation followed by a sustained release to maintain effective growth factor levels. As a result, the wound experiences enhanced granulation tissue formation, re-epithelialization, and matrix remodeling. Compared with the free TGF-β3 treatment group, the composite hydrogel group showed superior wound healing effects, indicating that the hydrogel system not only protects the bioactivity of TGF-β3 but also enhances its biological efficacy. Scratch assay and Ki-67 immunohistochemistry results indicate that BP-QS/TGF-β3 notably enhances fibroblast migration and proliferation. Moreover, the three-dimensional porous network of the hydrogel provides a favorable scaffold for cell migration, while the sustained release of TGF-β3 directly activates signaling pathways (e.g., Smad) to promote cellular proliferation and differentiation.

Although this study evaluated the biocompatibility and systemic toxicity of the hydrogel, the effect of this material on local macrophage polarization (M1/M2 phenotypes) was not evaluated in detail. The study did not investigate the expression profiles of inflammatory cytokines, including TNF-α, IL-1β, and IL-10, in the wound. Understanding the immunomodulatory functions of the hydrogel is anticipated to enhance comprehension of its wound healing mechanism. Regulating the immune microenvironment is a crucial strategy in diabetic wound treatment. Research indicates that immunomodulatory biomaterials significantly enhance the wound microenvironment and promote healing. A self-healing hydrogel containing exosomes sourced from bone marrow-derived mesenchymal stem cells has been shown to enhance angiogenesis and decrease inflammation by facilitating the shift of macrophages from the M1 to M2 phenotype (Lin et al., 2022; Zhao et al., 2023; Mohsin et al., 2024). Similarly, the potential of an autologous biogel enriched with antioxidants to promote diabetic wound healing was demonstrated by reshaping inflammatory patterns and restoring microenvironmental homeostasis (Yang et al., 2022b). To further promote diabetic wound healing, recent research has explored various novel therapeutic strategies. A photodynamic hydrogel targeting aldehyde dehydrogenase 2 (ALDH2) effectively reduced neutrophil extracellular trap formation, alleviated chronic inflammation, and facilitated M1-to-M2 macrophage polarization. This method improved tissue regeneration and stimulated angiogenesis by activating endothelial cells (Xiong et al., 2025). An adaptable injectable nanocomposite hydrogel derived from gelatin demonstrated multifunctional therapeutic effects, including antibacterial activity, oxidative stress reduction, and anti-inflammatory properties, by modulating the wound immune microenvironment, presenting a promising treatment strategy for chronic diabetic wounds (Yang et al., 2025). Future studies should therefore focus on exploring the immunoregulatory mechanisms of the BP-QS/TGF-β3 hydrogel, particularly its influence on macrophage polarization and cytokine dynamics, to fully exploit its therapeutic potential in diabetic wound management.

While this study systematically highlights the BP-QS/TGF-β3 composite hydrogel’s effectiveness in enhancing diabetic wound healing, it is important to recognize and address several limitations in future research, including an incomplete rheological characterization of the hydrogel, a lack of direct visual evidence for its biocompatibility (e.g., live/dead staining), and unvalidated antibacterial efficacy within the animal model. The molecular mechanisms require further detailed exploration, considering that the pathophysiological processes in the STZ-induced type 1 diabetic mouse model differ from those in chronic wounds typically linked to human type 2 diabetes. Future studies should focus on a detailed investigation of the hydrogel’s immunomodulatory effects and address the absence of long-term safety and efficacy data.

## 5 Conclusions

This study presents a high-performing candidate material for the synergistic therapy of refractory diabetic wounds, while also elucidating an effective combinatorial strategy that integrates antibacterial-controlled release and regeneration. The BP-QS/TGF-β3 hydrogel successfully combined antimicrobial protection with active wound regeneration stimuli, demonstrating considerable clinical translational potential. Future research will focus on further exploring its molecular mechanisms in regulating macrophage polarization and immune microenvironment remodeling, along with conducting long-term efficacy and safety assessments in large animal models to advance its clinical application.

## Data Availability

The original contributions presented in the study are included in the article/supplementary material. Further inquiries can be directed to the corresponding authors.
